# Serum and fecal profiles of aromatic microbial metabolites reflect gut microbiota disruption in critically ill patients: a prospective observational pilot study

**DOI:** 10.1186/s13054-020-03031-0

**Published:** 2020-06-08

**Authors:** Ekaterina Chernevskaya, Natalia Beloborodova, Natalia Klimenko, Alisa Pautova, Dmitrii Shilkin, Vitaliy Gusarov, Alexander Tyakht

**Affiliations:** 1 Federal Research and Clinical Center of Intensive Care Medicine and Rehabilitology, 25-2 Petrovka str., Moscow, Russia 107031; 2 Atlas Biomed Group - Knomics LLC, 31 Malaya Nikitskaya str., Moscow, Russia 121069; 3 Center for Precision Genome Editing and Genetic Technologies for Biomedicine, Institute of Gene Biology Russian Academy of Sciences, 34/5 Vavilova str., Moscow, Russia 119334; 4 N. Pirogov National Medical Surgical Center, 70 Nizhnyaya Pervomayskaya str., Moscow, Russia 105203

**Keywords:** Human gut microbiome, Critically ill patients, Metabolome, Aromatic microbial metabolites, GC-MS, ICU

## Abstract

**Background:**

High serum levels of certain aromatic microbial metabolites (AMM) are associated with severity and mortality in critically ill patients. Omics-based studies suggest gut dysbiosis and reduced microbiome diversity in critical conditions. However, the landscape of gut microbial metabolites is still to be outlined, not to mention the interplay correlation between the metabolome and gut microbiome in critically ill patients. The aim of this study was to analyze the association between serum and fecal levels of AMM and compare them with the composition of gut microbiota in critically ill patients in the acute and chronic stages.

**Methods:**

In this prospective observational pilot study, we analyzed the temporal dynamics of the gut microbiome and the AMM spectrum across two distinct subgroups—acute critical ill (ACI) patients with nosocomial pneumonia and chronically critically ill (CCI) patients (9 subjects each group)—as well as performed comparison with 23 healthy volunteers. The AMM levels for each patient were measured using GC-MS in simultaneously taken serum and fecal samples (SFS). These parameters were compared with 16S rRNA fecal microbiome profiles.

**Results:**

The observed proportions of bacterial taxa suggest a significant gut dysbiosis in the ACI and the CCI patients. Stronger imbalance in microbiome composition and dynamics observed in the ACI patients compared to the CCI ones resonates with a higher severity in the former group. The total levels of AMM in serum samples were higher for the ACI patients than for the CCI patients (3.7 (1.4–6.3) and 1.1 (1.0–1.6) μM, respectively; *p* = 0.0003). The qualitative composition of the SFS was also altered. We discovered significant associations between gut microbial taxa levels and metabolite concentrations in blood serum as well as in feces in each of the ACI and the CCI patients.

**Conclusions:**

Aromatic microbial metabolite profiles in the gut and the serum are interlinked and reflect a disruption of the gut microbial community in critically ill patients.

## Introduction

The importance of studying the qualitative and quantitative composition of gut microbiota in critically ill patients has been supported by a number of studies in recent years [1, 2]. The 16S rRNA sequencing microbiome assays allow us to assess the total prokaryotic community including uncultivable species in such patients. Dramatic changes in microbiota composition are caused primarily by hypoxia, hypercapnia, administration of drugs to suppress gastric secretion, vasoactive drugs, sedation, and analgesics which compromise the motility of the gastrointestinal tract, and cause a deficiency of nutrients in the intestine due to parenteral and enteral nutrition that deprives the microbiome of nutrients—for example, microbially accessed indigestible carbohydrates—and, of course, the use of antibiotics [3]. The “critical state microbiome” was characterized by an imbalance of commensal (“health-maintaining”) and opportunistic pathogenic bacteria, which made patients more susceptible to nosocomial infections, sepsis, and organ failure [4]. For example, the decay of commensal bacteria, mostly anaerobic, can lead to an overgrowth of *Enterococcus* [5] which is correlated with organ failure, length of stay in ICU, and mortality [2]. Despite the fact that no single reference structure for the gut community is known to date, there appears to be a functional homeostasis for a well-functioning microbiota “organ” in healthy individuals, which is directly related to metabolic balance in the human organism [6]. More than 30% of metabolites in the human body originate from gut microbiota and may contribute to host disease risk [7].

Among different classes of metabolites produced by members of human gut microbiota, investigation of aromatic microbial metabolites (АММ) is of particular interest [8]. Our experimental and clinical studies confirmed the biological activity of АММ and their involvement in the pathogenesis of bacterial infections. These substances affect the functions of mitochondria by producing active oxygen species, decreasing the rate of oxidation of NAD-dependent substrates [9], and suppressing the phagocyte activity of neutrophils in vitro [10]. The effects observed in vitro caused by the action of the AMM were similar to those in septic patients, and an increase in the concentration of these metabolites has been proposed as one of the causes of mitochondrial dysfunction in sepsis [11]. The following AMM are potentially involved in the pathological process: phenyllactic acid (PhLA), phenylpropionic acid (PhPA), phenylacetic acid (PhAA), *p*-hydroxyphenylacetic acid (*p*-HPhAA), and *p*-hydroxyphenyllactic (*p*-HPhLA) acid. АММ levels reflect the severity of the bacterial inflammatory process: they increase in patients with a local proinflammatory disease and reach a maximum level during severe sepsis and septic shock [12]. Our hypothesis regarding the bacterial origin of АММ was supported by the results obtained with isolates of clinically significant species of bacteria [13]. For example, *Staphylococcus aureus*, *Klebsiella pneumoniae*, and *Escherichia coli* produce PhLA and *p*-HPhLA, while non-fermenting (aerobic) Gram-negative bacteria (including *Acinetobacter* and *Pseudomonas*) are capable of forming *p*-HPhAA [14]. In microbial communities, phenylcarboxylic acids (PhCA) play an important role in the mechanisms of interspecies competition. PhPA and *p*-HPhAA, metabolites of human gut anaerobic bacteria, suppress the growth of *E. сoli* and *S. aureus* [15]. PhLA and *p-*HPhLA suppress the growth and reproduction of fungi [16]. In a healthy individual, metabolites containing lactic acid residue (PhLA and *p-*HPhLA) are not detected in the feces because they undergo deep biodegradation to end products under the action of anaerobic bacteria [17]. Given that АММ are involved in the mechanisms of interspecies interactions of microorganisms, a prolonged imbalance of АММ in the interior environment of a host might affect the composition and metabolic activity of gut microbiota and, as a result, disrupt the equilibrium between the macro- and microorganisms. The anaerobic bacteria of gut microbiome are thought to take part in the metabolism of phenylcarboxylic acids of endogenous origin: the suppression of metabolic activity of the microbiota might promote the accumulation of AMM [18]. A GC-MS analysis of 187 low molecular weight metabolites in the blood of 239 critically ill patients identified seven metabolites including *p-*HPhLA to be the most predictive of a 28-day lethality [19].

However, no data is currently available either on the production of AMM by the gut microbiota in critically ill patients or on their associations with microbial taxa in vivo. Moreover, the dynamics of microbiota in chronically critically ill patients who survive in acute critical illness and with prolonged dependence on mechanical ventilation and other intensive care therapies are yet to be studied. Meanwhile, сhronic critical illness is a serious and growing problem for healthcare systems: mortality exceeds that for most malignancies, and functional dependence on medical care persists for most survivors [20].

Thus, the study of metabolic activity and the identification of links between qualitative and quantitative characteristics of the microbiota of critically ill patients is highly relevant. The aim of this study was to analyze the association between the serum and fecal levels of AMM and compare them with the composition of the gut microbiota in critically ill patients in the acute and chronic stages.

## Materials and methods

### Study design and sample collection

This study was approved by the institutional review board of the N. Pirogov National Medical Surgical Center and the local ethics committee of the Federal Research and Clinical Center of Intensive Care Medicine and Rehabilitology. It was conducted in the ICU of the two abovementioned centers from February 2016 to May 2017. Formal consent for participation in this study was obtained from each patient or his/her legal representative.

Forty-seven pairs of serum and fecal samples (SFS) were simultaneously collected from critically ill patients: 24 SFS from 9 patients with acute critical ill (ACI) patients  with nosocomial pneumonia (3 (3, 4) days after admission to ICU), 23 SFS from 9 chronically critically ill (CCI) patients in the course of prolonged neurorehabilitation (90 (67–100) days after admission to ICU) after different forms of acquired brain injury such as traumatic brain injury or stroke. Blood (from a central venous catheter) and gut microbiome samples were collected in the morning at regular intervals: in ACI patients—on days 1, 3, and 7–9 after the diagnosis of pneumonia; in CCI patients—once a week for a month. To compare serum metabolite levels, we used serum samples from 23 healthy volunteers (for 5 of them, fecal samples were also collected on the same day). Microbiome samples were obtained by collecting a small amount of feces as a rectal swab and dissolving it in 1 ml of sterile saline solution; after thorough mixing, it was divided into two Eppendorf tubes. All samples were frozen and stored at − 30°С prior to analysis which included GC-MS, biomarkers, and general clinical analyses and laboratory tests. The baseline clinical and laboratory characteristics of the critically ill patients are shown in Table 1. All patients received high-calorie fiber-free enteral tube feeding; in the ACI group, the enteral nutrition was initiated within 48 h of admission. None of the patients received renal replacement therapy. Most patients required some form of a respiratory support and antibiotic intake (data available in Supplementary Tables 1 and 2).

**Table 1:** Baseline clinical and laboratory characteristics of critically ill patients. Quantitative data are shown as median (1st and 3rd quartile); reference values are provided for PCT and WBC; *ns* not significant

Parameter	ACI	CCI	***p*** value
Age	58 (47–67)	45 (20–70)	ns
SOFA	5 (3–6)	4 (4–5.5)	ns
BMI	26 (24.0–36.2)	21.8 (20.7–24.3)	*< 0.05*
PCT (0.25 ng/mL)	1.84 (0.39–2.00)	0.04 (0.02–0.08)	*< 0.05*
WBC (4–9 × 10^9^/L)	12.7 (9.6–15.1)	9.6 (7.6–10.6)	ns
Inotropes	5/9	1/9	ns

*SOFA*Sequential Organ Failure Assessment,*PCT*procalcitonin,*WBC*white blood cells,*BMI*body mass index

### Fecal sample preparation and microbiome data analysis

Fecal DNA extraction, sequencing library preparation for the 16S rRNA gene (V2, V3, V4, V6–7, V8, and V9 regions), and sequencing were performed on an Ion Torrent Personal Genome Machine (PGM) platform (Life Technologies; Carlsbad, CA, USA) using an Ion 16S Metagenomics Kit (Life Technologies; Carlsbad, CA, USA) as previously described [21].

Data analysis was performed in the Knomics-Biota system [22] and manual R scripts (interactive reports and the raw reads are publicly available online at https://biota.knomics.ru/amm-and-gut-microbiome-2019). Reads were analyzed using QIIME 1.9.1 [23]. They were trimmed to remove bases with Phred scores lower than 20, then reads shorter than 75% of the initial length were discarded. Reads were classified using a closed-reference OTU picking (vsearch algorithm [24]) against a 16S rRNA sequence database (Greengenes v. 13.5 [25], 97% OTU similarity). The classified reads for each sample were randomly rarefied to even coverage (5000 reads per sample). Read counts of microbial species, genera, and families were calculated as the sum of reads classified as OTUs belonging to the respective taxon. The relative abundance of each taxon was calculated by dividing the number of its rarefied reads by the total read count for the sample. The alpha diversity of the communities was estimated via the Shannon index at the level of OTUs.

### Standards and reagents

Benzoic acid (BA, ≥ 99.5%), 2,3,4,5,6-D_5_-benzoic acid (internal standard, ≥ 99 atom % D, ≥ 99%), phenylpropionic acid (PhPA, ≥ 99%), phenyllactic acid (PhLA, ≥ 98%), 4-hydroxyphenylacetic acid (*p*-HPhAA, ≥ 98%), homovanillic acid (HVA, ≥ 97%), 4-hydroxyphenyllactic acid (*p*-HPhLA, ≥ 97%), 3,4-dihydroxybenzoic acid (internal standard, ≥ 98%), 4-hydroxyphenylpropionic acid (*p*-HPhPA, ≥ 98%), 4-hydroxybenzoic acid (*p*-HBA, ≥ 99%), N,O-bis (trimethylsilyl) trifluoroacetamide (BSTFA, contains 1% trimethylchlorosilane, 99% BSTFA), and hexane (≥ 97.0%) were obtained from Merck (Germany); the sulfuric acid, acetone, diethyl ether, and sodium chloride were laboratory reagent grade and obtained from Khimreactiv (Russia).

### GC-MS analysis

Serum samples were thawed at room temperature prior to use. All GC-MS analyses were performed on a Trace GC 1310 gas chromatograph equipped with an ISQ LT mass spectrometer using a TR-5MS capillary column (95% poly (dimethylsiloxane) + 5% phenyl polysilphenylene-siloxane phase, 30 m × 0.25 mm, df = 0.25 μm) obtained from Thermo Scientific (Thermo Electron Corporation, USA). The column flow was constant at 1.5 mL min^−1^ with helium as the carrier gas, split 1:20. The GC analysis was performed in 27 min with a starting oven temperature of 80 °С (hold time 4 min), a ramp of 10 °С min^−1^ to 250 °C and a ramp of 20 °С min^− 1^ to 280 °С (hold time 5 min). The inlet temperature was 200 °С, and the injection volume was 1 μL. Full-scan mass spectra were recorded with a *m/z* range of 50–480 in the electron-impact mode at 70 eV, using Xcalibur 2.2 software. The MS source was 200 °С and the GC-MS interface was kept at 250 °С. The scan rate was 3 scans/s; the cathode delay time was 4 min. Trimethylsilyl derivatives of the PhCAs were identified using retention times and characteristic *m/z*. Mass spectral data for the trimethylsilyl derivatives of the PhCAs were proved by the NIST mass spectrum library. The retention times and characteristic *m/z* values of trimethylsilyl derivatives of the PhCAs have been described in detail in our previous study [26].

Quantitative analyses of the AMM in serum samples were carried out using the following internal standards: 2,3,4,5,6-D_5_-benzoic acid for BA and PhPA; 3,4-dihydroxybenzoic acid for PhLA, *p*-HBA, HVA, *p*-HPhAA, *p*-HPhPA, and *p*-HPhLA. The concentrations of the AMM were calculated using the calibration curve equations described in our previous study [26].

Quantitative analyses of the AMM in fecal samples were not performed due to sample heterogeneity. The results on the PhCA in fecal samples were illustrated as the proportion of each acid among all AMM (%).

### Liquid-liquid extraction from serum samples

The conditions for liquid-liquid extraction of the AMM have been previously described [26]. Briefly, a 200-μL aliquot of serum and a 100-μL aliquot of aqueous solution of internal standards (7.5 mg L^−1^) were diluted with 800 μL of distilled water. Solid sodium chloride (0.3–0.5 g) and a 15-μL aliquot of concentrated sulfuric acid were added. An extraction with diethyl ether  was carried out (2 × 1 mL). The ether extract was evaporated at 40 °С and derivatized with BSTFA (40 μL, 90 °С, 30 min). The solution was cooled at 5 °С for 30 min and diluted with 400 μL of *n*-hexane, and 1 μL of the final solution was injected into the GC-MS system.

In the case of the feces, a solid sample (usually in 1.5 mL Eppendorf) was diluted in 1 mL of distilled water and vortexed for 2 min and a 200-μL aliquot of the solution was subjected to the described sample preparation protocol for the serum.

### Statistical analysis

Quantity concentration values were calculated for serum metabolite levels using the previously described method [26]. For fecal metabolites, absolute quantitative concentrations could not be reliably obtained. Therefore, the levels of metabolites in the feces were converted into relative values proportionally in a way that for each sample, the total levels of all measured metabolites in the feces were equal to one.

A between-group comparison of microbial taxa abundances was performed for arcsin-sqrt transformed abundance values at each taxonomic level. The significance of the associations was evaluated using a mixed-effect model, where the patient identifier was included as a random effect. An adjustment for multiple comparisons was carried out using the Benjamini-Hochberg method separately at each taxonomic level. The same approach was applied for the analysis of the associations between taxa abundances and levels of metabolites in the serum and the feces. Only the taxa with transformed abundance levels of > 0.002 in > 5 samples and only the metabolites with non-zero values in > 5 samples were considered in these analyses.

A similar mixed-effect model with a patient identifier as a random effect was applied to investigate the alpha diversity between-group analysis and associations between alpha diversity and the level of metabolites, as well as for testing the statistical significance of correlations between the levels of metabolites in the blood and feces (along with the Pearson correlation coefficient). Only the metabolites with non-zero values in > 5 samples were considered here.

A microbe-metabolite graph was constructed based on the identified associations between the levels of metabolites in the blood and feces as well as between the levels of metabolites and abundance of bacterial genera. In addition, genera co-occurrence was assessed by applying a SPIEC-EASI algorithm to non-rarefied abundance tables [27]. Genera detected at < 10 reads per sample on average or in < 10 samples were excluded. In the SPIEC-EASI algorithm, neighbors were selected using the Meinshausen and Bühlmann method, and the model selection was performed using the StARS algorithm (huge R package [28]) (number of lambda iterations = 10, minimum lambda ratio = 0.3).

## Results

### Gut taxonomy dysbiosis in critically ill patients and healthy subjects

**Fig. 1:**
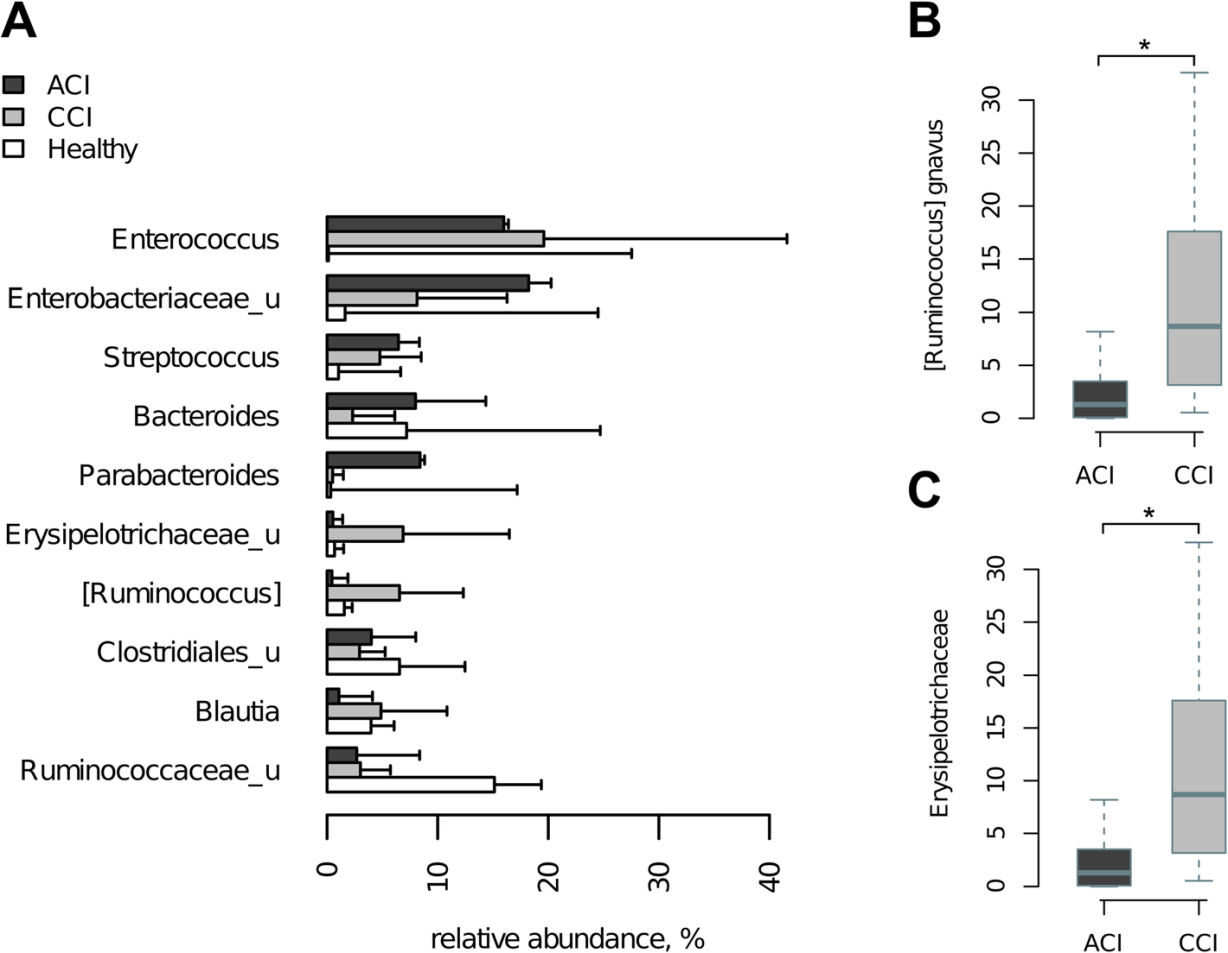
Predominant species of gut microbiome for two different groups of critically ill patients. **a** Major microbial genera in gut microbiome in each of the groups of patients: ACI (*N* = 24), CCI (*N* = 22), and healthy cohort from previous study (*N* = 215) [29]. **b**, **c** Taxa with significantly different abundance between CCI (*N* = 22) and ACI (*N* = 24) patients

The critical conditions of patients are partially reflected in their gut microbial community structure (genus-level composition is shown in Supplementary Figure 1, 2; the detailed interactive reports are available online at https://biota.knomics.ru/amm-and-gut-microbiome-2019). The proportions of bacterial taxa in each group suggest a significant dysbiosis (Fig. 1). As a major feature, there is a strong enrichment in microbes associated with inflammation—*Enterobacteriaceae*, *Enterococcus*, *Streptococcus*, and *Staphylococcus*, coupled with a depletion of commensal microbes. Their relative abundance does not typically exceed 1% in healthy subjects (as estimated in the same population using similar approaches) [29–31].

Microbiome compositions were different between the ACI and CCI groups. Particularly, the *[Ruminococcus] gnavus* species and *Erysipelotrichaceae* family were more abundant in the CCI group than in the ACI group (adj. *p* = 0.01 and 0.06, respectively; Fig. 1); for both taxa, these differences were also reflected at higher taxonomic ranks.

Unlike the CCI group, the ACI group included non-survivors (in total 7 survivors and 2 non-survivors). Due to the imbalance between the two survival categories and small sample size, a reliable comparison between them could not be performed. Noteworthy, a selection of Gram-positive microorganisms was observed in non-survivors: one patient on the first day had an extremely high level of *Staphylococcus aureus* (70.9%) compared with other patients (less than 0.1%); the other patient had more than 40% *Streptococcus* spp. in the fecal sample.

**Fig. 2:**
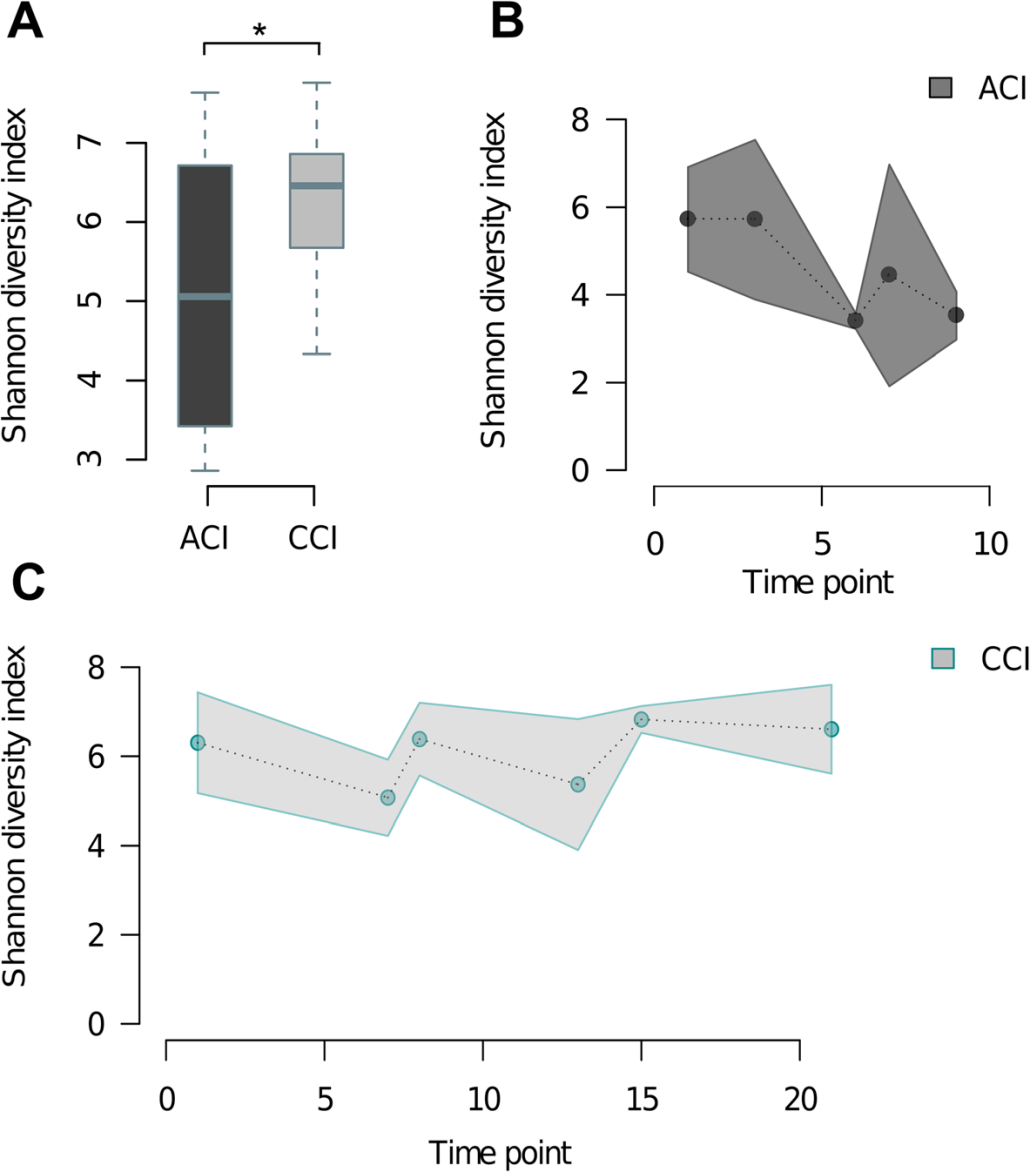
Alpha diversity (richness) of the gut microbiome in groups. **a** Pooled within each group. **b** Temporal dynamics in the ACI group. **c** Temporal dynamics in the CCI group (the dotted line connects mean daily values of diversity, and the solid lines indicate the standard deviation from the mean). *p < 0.05, mixed-effect linear model

The alpha diversity of the gut microbial community was lower in the ACI group compared to that in the CCI group (5.2 ± 1.7 vs. 6.2 ± 1.0, Shannon index, *p* = 0.0627; Fig. 2a). The mean value of the Shannon diversity index in the healthy population estimated in the previous study was 6.2 ± 0.6 [29]. The dynamics of alpha diversity during hospitalization are shown in Fig. 2b, c. While it sharply decreased in the ACI group, it remained relatively stable in the CCI one.

Overall, the observed stronger imbalance in the gut microbiome composition and dynamics in the ACI patients than in the CCI group corresponds with a higher severity in the former group.

### Aromatic metabolite profiles in critically ill patients and healthy subjects

**Fig. 3:**
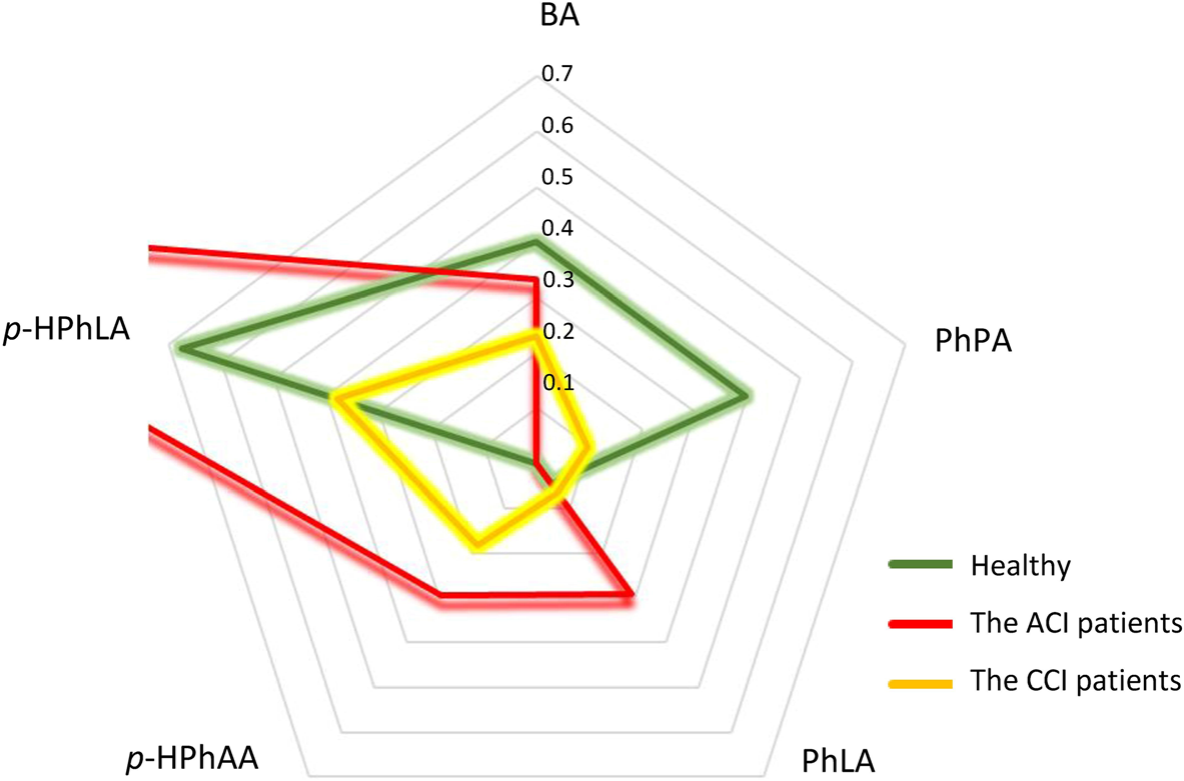
Quantitative profiles of the AMM (median, μM) in serum samples of three groups: healthy subjects, the ACI patients, and the CCI patients. Note: the outlying level of *p-*HPhLA in the ACI group is 1.4 μM. BA benzoic acid, PhPA phenylpropionic acid, PhLA phenyllactic acid, *p*-HPhAA *p*-hydroxyphenylacetic acid, *p*-HPhLA *p*-hydroxyphenyllactic acid

The summary levels of microbial metabolites (∑AMM) in the serum samples were higher for the ACI patients than for the CCI patients (3.7 (1.4–6.3) and 1.1 (1.0–1.6) μM, respectively; *p* = 0.0003). For each of the groups, the distribution was different than in samples of the healthy subjects—2.0 (1.4–2.3) μM (*p* < 0.05) (Fig. 3).

**Fig. 4:**
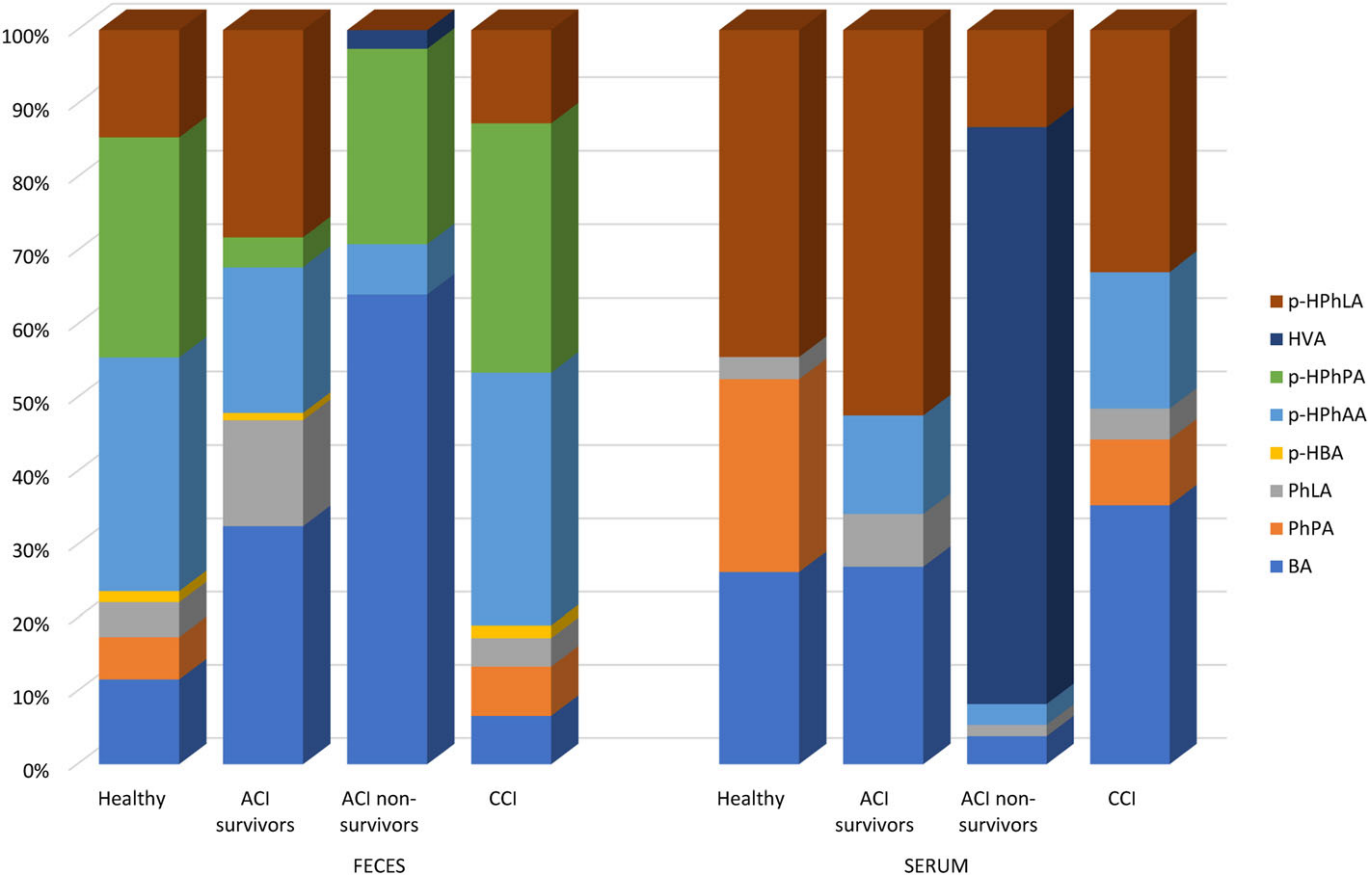
Comparison of the AMM qualitative profiles in serum and feces. The AMM profiles in feces and serum from the healthy subjects (*n* = 23), the ACI patients (survivors, *n* = 7; non-survivors, *n* = 2), and the CCI (*n* = 9) patients. The data are presented as the median within-group proportion of each acid among all AMM. BA benzoic acid, PhPA phenylpropionic acid, PhLA phenyllactic acid, *p*-HBA *p-*hydroxybenzoic acid, *p*-HPhAA *p*-hydroxyphenylacetic acid, *p*-HPhPA p-hydroxyphenylpropionic acid, HVA homovanillic acid, *p*-HPhLA *p*-hydroxyphenyllactic acid

The qualitative composition of SFS was also different compared to the healthy subjects (Fig. 4). We observed a prevalence of BA, *p-*HPhLA, *p-*HPhPA, and *p-*PhAA in the ACI group with a higher level of BA in the gut and HVA in the serum of non-surviving patients and a predominance of hydroxylic acids (*p-*HPhAA and *p-*HPhPA) in the CCI samples. On the contrary, the SFS of the healthy subjects were characterized by an absolute prevalence of PhPA as well as by low (around 2 μM) levels of all AMM.

Overall, the serum as well as the fecal AMM profiles appeared to be disrupted in critically ill patients, with a more pronounced alteration for the ACI group and especially in non-survivors (Fig. 4).

### Metabolic correlations between feces and blood in the ACI and the CCI groups

**Fig. 5:**
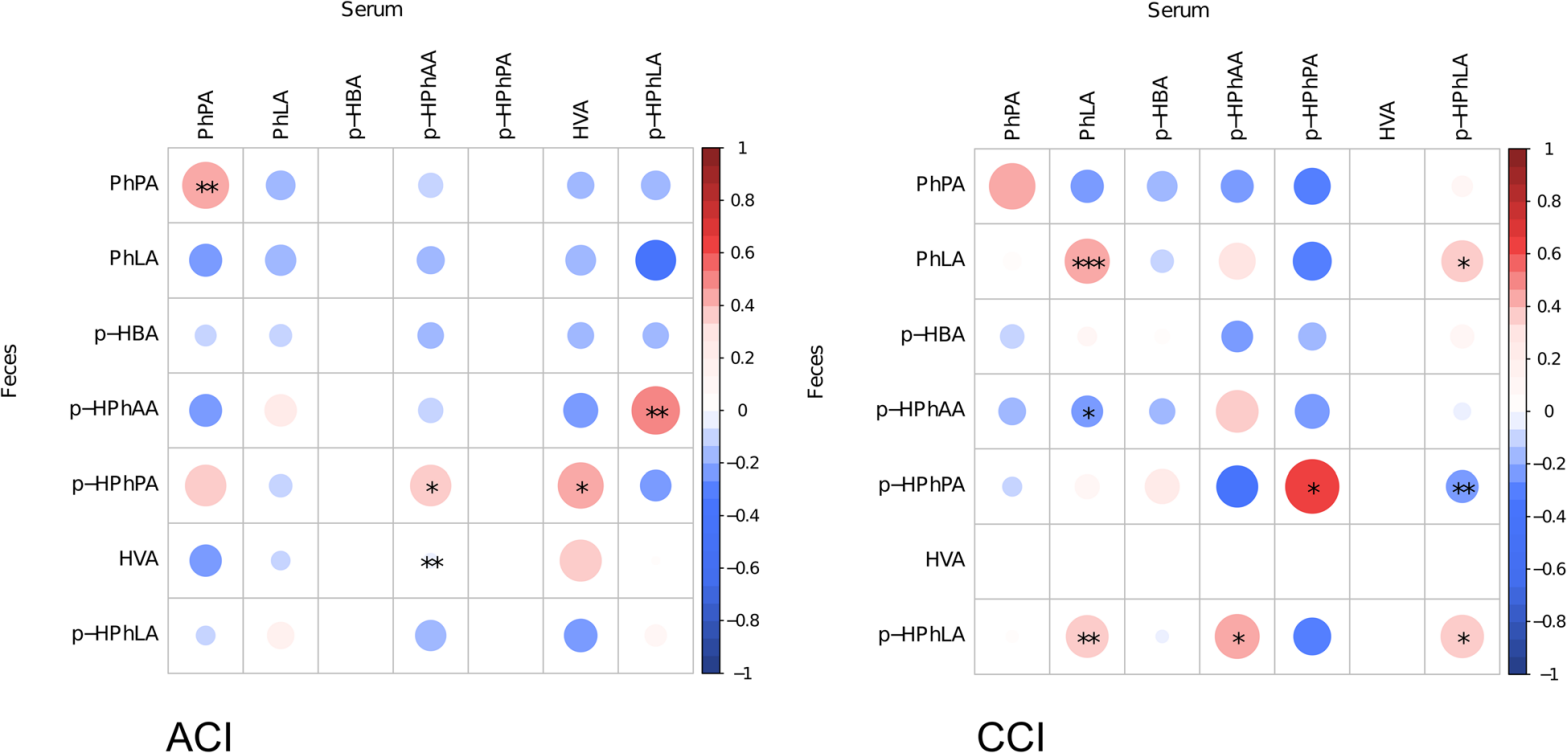
Correlations between aromatic metabolite levels in feces and serum. The size of circles denotes the absolute value of the Pearson correlation coefficient between metabolite levels; blue colors show negative and red positive correlations. Asterisks denote significant associations (***raw *p* < 0.01, **raw *p* < 0.05) and trends (*raw *p* < 0.1). PhPA phenylpropionic acid, PhLA phenyllactic acid, *p*-HBA *p-*hydroxybenzoic acid, *p*-HPhAA *p*-hydroxyphenylacetic acid, *p*-HPhPA *p*-hydroxyphenylpropionic acid, HVA homovanillic acid, *p*-HPhLA *p*-hydroxyphenyllactic acid

We compared the levels of all AMM between feces and serum in a paired way (Fig. 5). There were only a few metabolites for which these levels were correlated. In the CCI group, a positive same-metabolite correlation was observed for PhLA (Pearson correlation, *r* = 0.4232), *p-*HPhPA (*r* = 0.6153), and *p-*HPhLA (*r* = 0.3905). In the ACI group, a similar effect was observed only for PhPA (*r* = 0.4682); noteworthy, the correlation patterns did not overlap between the two groups of patients.

There were also several associations between feces and serum levels of distinct metabolites. In the CCI group, the *p-*HPhLA level in the feces correlated with the levels of two metabolites in the serum—PhLA (*r* = 0.3796) and *p-*HPhAA (*r* = 0.4182). The PhLA level in feces also positively correlated with *p-*HPhLA in serum (*r* = 0.3620). In the ACI group, the *p-*HPhPA level in the feces correlated with HVA (*r* = 0.4392) and *p-*HPhAA (*r* = 0.3517) in serum. Additionally, in the ACI group, the *p-*HPhAA in the feces strongly correlated with *p-*HPhLA in the serum (*r* = 0.4880) (the opposite observation was made in the CCI group—i.e., for *p-*HPhAA in the serum vs. *p-*HPhLA in the feces).

### Microbiome-metabolome interactions in the ACI and the CCI groups

**Fig. 6:**
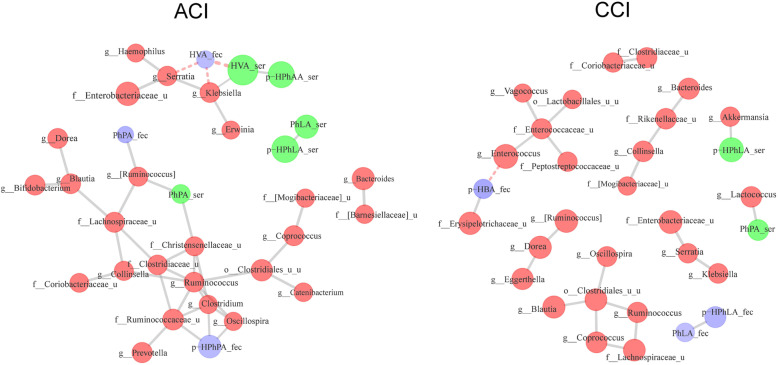
Microbe-metabolite interactions in critically ill patients. In the graphs, the nodes are bacterial genera (red) and metabolites in blood (green) and feces (blue). The edges denote significant positive (gray-colored) and negative (red-colored, dotted) associations

We examined associations between and within three types of measurements: microbial genera levels and metabolite concentrations in blood serum and in feces. This analysis was conducted separately for the ACI and for the CCI group. Healthy control samples were not included due to the lack of microbiome data. The associations are summarized on the graph (Fig. 6).

The analysis of the clusters of co-occurring microbes and correlations of these clusters with metabolites revealed interesting differences between the ACI and the CCI groups. One of the microbial clusters similar between the two groups included opportunistic pathogens: *Klebsiella*, *Serratia*, and unclassified *Enterobacteriaceae*. In the CCI group, the cluster was not associated with any of the metabolites neither in the serum nor in the feces. However, in the ACI group, it had positive associations with HVA and *p-*HPhAA in the serum and negative with HVA in the feces. As shown above, high serum HVA levels were revealed for non-survivors in the ACI group. Previously, we have observed increased HVA and *p-*HPhAA levels in patients with septic shock [32].

In the CCI group, there was another microbial cluster including gut opportunists *Enterococcus*, unclassified *Enterococcaceae*, and *Peptostreptococcus* that was negatively associated with *p*-HBA in feces.

Both groups of patients also included a number of co-occurring bacterial clusters previously observed in healthy gut microbiota [29, 31]. The largest cluster in the ACI group included commensal microbes: unclassified *Lachnospiraceae*, *Clostridiaceae* and *Coriobacteriaceae*, *Ruminococcus*, *Blautia*, *Oscillospira*, and *Dorea*. The same bacteria formed several distinct clusters in the CCI group. However, the cluster had significant associations with metabolite levels only in the ACI group—the positive associations with PhPA levels in feces and serum and *p-*HPhPA levels in feces.

The described graphs include associations between bacterial genera and metabolites. The associations between all taxonomic level taxa and metabolite levels are additionally listed in Table 2.

**Table 2:** Significant associations between metabolites and bacteria for ACI and CCI patients (FDR adjusted *p* < 0.1)

Metabolites	Group	Source	Association	Bacterial taxon
PhPA	CCI	Serum	+	*Lactococcus*
	ACI	Serum	+	*[Ruminococcus] gnavus* *[Ruminococcus]* *Unclassified Christensenellaceae* *Christensenellaceae* *Collinsella aerofaciens*
		Feces	+	*[Ruminococcus]* *[Eubacterium] dolichum*
*p-*HPhPA	ACI	Feces	+	*Ruminococcaceae unclassified* *Ruminococcaceae* *Veillonellaceae* *Clostridium* *Oscillospira unclassified* *Oscillospira* *Lactobacillus unclassified* *[Eubacterium] dolichum*
			−	*Bacteroides unclassified*
PhLA	CCI	Feces	−	*Deltaproteobacteria*
	ACI	Serum	+	*Ruminococcus*
		Feces	+	*Prevotella copri*
*p-*HBA	CCI	Serum	+	*Proteobacteria*
		Feces	+	*Erysipelotrichaceae*
			−	*Enterococcus*
HVA	ACI	Feces	−	*Serratia marcescens* *Serratia* *Klebsiella unclassified* *Klebsiella*
		Serum	+	*Serratia marcescens* *Klebsiella*
*p-*HPhAA	ACI	Feces	+	*Parabacteroides*
*p-*HPhLA	CCI	Serum	+	*Akkermansia*
		Feces	−	*Deltaproteobacteria*

Overall, the number of discovered associations between microbiome composition and metabolites was lower for the CCI group. For some metabolites (*p*-HPhPA, HVA, and *p*-HPhAA), the associations were only observed in the ACI group, while for *p*-HPhLA and *p*-HBA, only in the CCI group. The associations with microbes were quite unique between serum and fecal metabolite levels. The only common association was *[Ruminococcus]-*positive correlation with PhPA in the ACI group. Interestingly, some of the associations had opposite directions for metabolites measured in feces and serum: the HVA serum level in the ACI group was positively associated with some proinflammatory bacteria (*Serratia*, *Klebsiella*) in the serum and negatively, in the feces.

In addition to the analysis of individual taxa, the alpha diversity of the microbial community was also compared with AMM concentrations. We detected positive association between alpha diversity and *p-*HPhPA fecal level in the ACI group (Shannon diversity index, FDR = 0.0017).

## Discussion

In this prospective observational pilot study, taxonomic and metabolic characteristics of gut microbiota were evaluated in critically ill patients and healthy subjects. The dynamics of the metagenome were compared between the ACI and CCI patients to shed light on the evolution of the microbial community in the ICU during transition from acute to chronic state. We discovered a pronounced dysbiosis with a prevalence of proinflammatory taxa and a reduced diversity in both groups of patients—in agreement with the results of previous studies [1, 2]. There were profound changes in the qualitative and quantitative profiles of AMM in comparison with the healthy subjects. The major findings of this study were associative patterns between clusters of microbial taxa and aromatic metabolite levels (in serum and feces).

We observed certain differences in baseline microbiome composition of the ACI and CCI groups of patients. The CCI group had higher relative abundance of *Erysipelotrichaceae* family and *[Ruminococcus] gnavus* species—opportunists that could contribute to the chronic illness severity. The members of *Erysipelotrichaceae* appear to be highly immunogenic and can potentially overgrow in the gut after treatment with broad-spectrum antibiotics [33–35]. Multiple studies associate the enrichment of the mucus-dwelling [*Ruminococcus] gnavus* taxon with inflammatory bowel diseases [36–38] (in our cohort, it was about an order of magnitude greater than reported in healthy subjects). These findings, along with the overall unstability of the patients’ microbiota (Supplementary Figs. 1 and 2), will be validated in our further studies with a higher number of subjects in each group.

We observed qualitative and quantitative imbalance of microbial metabolites in two groups of patients in comparison with healthy subjects. The profile of AMM in the CCI group can be described as “less and mess” (a low quantity and modified composition) characterized by a significantly lower concentration of PhPA and sum of AMM in comparison with the healthy subjects. At the same time, the summary levels of microbial metabolites in the serum samples were higher for the ACI patients. Earlier, we have shown that the sum of three AMM (*p-*HPhAA, *p-*HPhLA, PhLA) reflects the severity of disease in patients admitted to ICU as good as conventional scales APACHE II and SOFA [32, 39] and their concentrations increased in dynamics in non-survived patients [26], due to their production by pathobionts, such as *Staphylococcus aureus*, *Klebsiella pneumonia*, *Acinetobacter baumanii*, *Pseudomonas aeruginosa*, and *Escherichia coli* [40, 41].

Remarkably, we discovered that associations between microbial taxa clusters and metabolite concentrations in blood serum and feces varied between the ACI and CCI groups. In the ACI group (including patients with pneumonia), a significant positive correlation between serum and feces was observed for PhPA metabolite only. At the same time, in CCI patients (without any locus of infection), such link was observed for PhLA, *p-*HPhPA, and *p-*HPhLA metabolites. This suggests the existence of microbial networks involved in aromatic compound metabolism which undergo disruption in a critically ill host, and the degree of such disruption might vary in acute and chronic illness. Further studies based on genomic reconstruction of the respective pathways will elucidate the robustness of such networks and roles of particular species in their ecology.

Our hypothesis was that not only the gut might contribute to metabolic activity but also other sites of infection—for example, lungs, as in cases of ACI patients with nosocomial pneumonia. Accordingly, in the ACI group, the serum level of HVA is positively associated with *Klebsiella* and *Serratia* genera but these associations were negative for feces. HVA is a major catecholamine metabolite, a deaminated and o-methylated metabolite of dopamine [42]. The strong correlations between *p-*HPhAA and HVA and the presence of shock and also the possibility of bacterial biodegradation of *p-*HPhAA into inhibitors of catecholamine synthesis confirm the involvement of AMM in the pathogenesis of septic shock [32]. For example, *K. pneumoniae* [43], *P. aeruginosa* [44], and other Gram-negative species are capable of degrading *p-*HPhAA to 3,4-dihydroxymandelic acid (DHMA) and 3,4-dihydroxyphenylacetic acid (DOPAC), with the subsequent formation of HVA. The results of a previous pilot study suggested associations between endogenous catecholamines and features of the bacterial lung microbiome that contribute to the pathogenesis of respiratory infections [45]. The catabolism of these compounds in vitro followed by growth has been demonstrated for many microorganisms including *P. aeruginosa* [46, 47].

Interestingly, PhPA levels were close to undetectable in both groups. The PhPA deficiency was observed in various serious diseases including sepsis [41]. In our cohort, one of its precursors—*p-*HPhPA—has a positive correlation with Gram-positive bacteria (including *Christensenellaceae*, *Oscillospiraceae* families, and *[Ruminococcus]* genera) commonly representing a healthy microbiome (Table 2). Presumably, the presence of such bacteria allows maintaining a long-lasting adequate metabolism even in critical conditions. The *Christensenellaceae* and *Oscillospiraceae* families have previously been shown to include microorganisms with potential health benefits: they are associated with lower body mass index (BMI) [48] and contribute to the formation of secondary bile acids [49, 50].

One of the interesting associations which we found was between *Akkermansia* and *p-*HPhLA in the CCI patients. *A. muciniphila* is a mucin-degrading bacterium of the Verrucomicrobia phylum linked to metabolism in animals and humans due to its potential anti-inflammatory properties [51, 52]. However, the microbe has been also linked to proinflammatory pathways, playing an important role in neurodegenerative disease [53]—an intriguing fact in the context of our CCI group including neurorehabilitation patients. Although its relation to *p-*HPhLA metabolism has not been assessed, the ability to produce the compound (as well as PhLA) was demonstrated in vitro for some strains of the species *Lactobacillus* and *Bifidobacterium* species [14], while species of anaerobic bacteria (*Bacteroides fragilis*, *B. thetaiotaomicron*, *Clostridium perfringens*) were linked to reduced levels of this acid [18]. Further study is needed to evaluate the potential role of the *p-*HPhLA as a factor that can reflect the gut microbiota dysbiosis and whether extreme alterations in gut microbiota are associated with patient neurological disorders.

The present study has some limitations. Firstly, in this pilot project, the sample size is rather small and further studies will be conducted to validate the observed links of critical illness severity with the gut microbiota composition dynamics and metabolome. The antibiotic use was extensive, with the set of antibiotics and their classes widely varying from subject to subject. Although it did not allow a direct statistical adjustment for this factor, we did not observe distinct differences between the CCI and the ACI groups. Although all patients received similar enteral nutrition, in the ACI, the first sample was collected before its start. Also, inotropic support rate and BMI were different between the groups. Overall, together with different diagnoses, the observed heterogeneity for many factors is typical in ICU setting and may have confounded our results.

Our results highlight the complexity of metabolic interactions between the members of the gut microbial ecosystem, especially when the diversity declines dramatically in critical conditions. This concept can be well demonstrated by community-level microbial ecology models based on genomic metabolic reconstructions, where it is shown that, despite marked resource competition at the level of whole assemblies, microbial communities harbor metabolically interdependent groups which recur across diverse habitats. Cooperating groups can make efficient use of limited resources through metabolite exchange, providing a survival advantage and enabling coexistence in diverse niches [54]. We believe that understanding the metabolic language of microorganisms and moving from the concept of single pathogens to a holobiont will serve as a catalyst for the development of new disease prevention and treatment strategies based on a multi-omics approach.

Overall, there is a great challenge in improving clinical predictive models for critically ill patients using microbiome-related parameters. Its urgence is being supported by the currently developing situation of treating COVID-19 patients faced by clinicians in ICU worldwide: secondary bacterial infections are likely to worsen the outcomes [55], with some of the opportunists originating from the gut. Our results confirm that AMM are promising biomarkers that can be routinely measured using GC-MS to facilitate therapeutic decisions. The method advantages include simple sample preparation, cost-effectiveness, and short time of analysis (several hours). Additionally, although 16S rRNA analysis is yet to become rapid enough to be applicable in ICU, our findings highlighted clinically relevant biomarker taxa that can be analyzed using quicker tests like taxon-specific qPCR tests [56].

## Conclusion

We identified a distinct gut microbiome composition in chronically and acute critically ill patients, with the latter characterized by extremely low diversity. A relationship was found between the alterations of microbiome and changes in serum and fecal profiles of aromatic microbial metabolites. Variation of these associative patterns between two groups of the patients suggests different scenarios of microbiome imbalance-mediated contribution to the risks of disease severity. Analysis of aromatic metabolites combined with microbiome survey is a promising way for improving efficacy of surveillance and treatment in ICU.

## Supplementary information

**Additional file1 : Supplementary Figure 1.** - Temporal dynamics of gut microbiome composition in the ACI patients. The heatmap of relative abundance at genus level is split into sections by subject, with rows of each section corresponding to consecutive time points. A snapshot from an online interactive report in Knomics-Biota (https://biota.knomics.ru/amm-and-gut-microbiome-2019).

**Additional file 2: Supplementary Figure 2.** - Temporal dynamics of gut microbiome composition in the CCI patients. The heatmap of relative abundance at genus level is split into sections by subject, with rows of each section corresponding to consecutive time points. A snapshot from an online interactive report in Knomics-Biota.

**Additional file 3: Supplementary Table 1.** - Clinical characteristics of the patients in the ACI and the CCI groups of patients. **Supplementary Table 2.** - Additional sample information: patient ID and time point (day of collection).

## Data Availability

The datasets used and/or analyzed during the current study are available from the corresponding author on reasonable request.

## Abbreviations

ACI: Acute critically ill
AMM: Aromatic microbial metabolites
CCI: Chronically critically ill
GC-MS: Gas chromatography-mass spectrometry
ICU: Intensive care unit
OTU: Operational taxonomic unit
SFS: Serum and fecal samples
SIRS: Systemic inflammatory response syndrome
BA: Benzoic acid
HVA: Homovanillic acid
PhPA: Phenylpropionic acid
PhAA: Phenylacetic acid
PhLA: Phenyllactic acid
*p*-HPhLA: *p*-Hydroxyphenyllactic
*p*-HPhAA: *p*-Hydroxyphenylacetic acid
*p*-HPhPA: *p-*Hydroxyphenylpropionic acid
*p*-HBA: *p-*Hydroxybenzoic acid
