# Supplementary material for: Serum and fecal profiles of aromatic microbial metabolites reflect gut microbiota disruption in critically ill patients: a prospective observational pilot study
Source: Crit Care. 2020 Jun 8;24:312. doi: 10.1186/s13054-020-03031-0 (PMC7278238; doi:10.1186/s13054-020-03031-0)
Supplement: Supplementary file 1 — Additional file1 : Supplementary Figure 1. - Temporal dynamics of gut microbiome composition in the ACI patients. The heatmap of relative abundance at genus level is split into sections by subject, with rows of each section corresponding to consecutive time points. A snapshot from an online interactive report in Knomics-Biota (https://biota.knomics.ru/amm-and-gut-microbiome-2019). [file 13054_2020_3031_MOESM1_ESM.pdf]

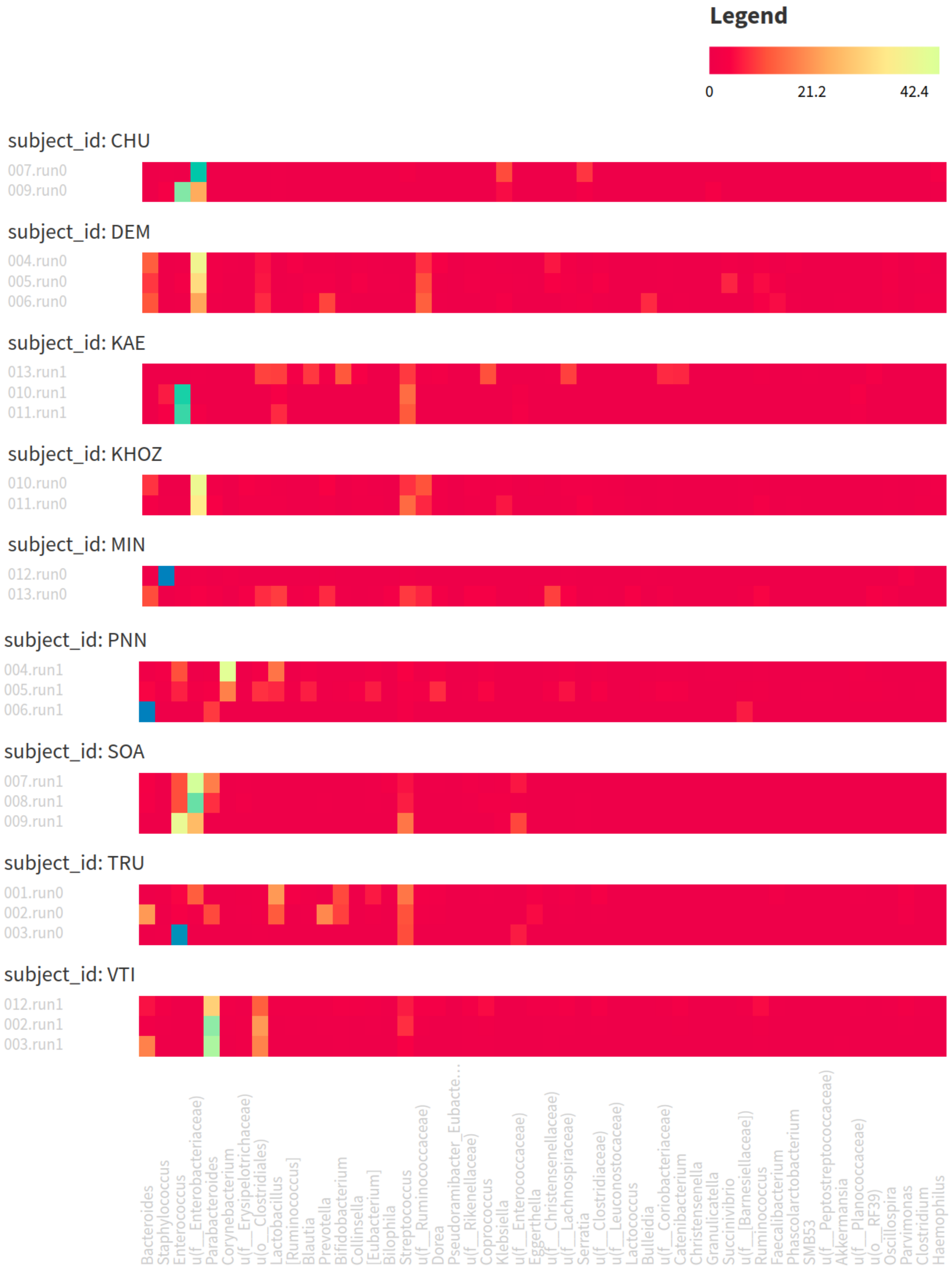

Legend
0
21.2
42.4
subject_id: CHU
007.run0
009.run0
subject_id: DEM
004.run0
005.run0
006.run0
subject_id: KAE
013.run1
010.run1
011.run1
subject_id: KHOZ
010.run0
011.run0
subject_id: MIN
012.run0
013.run0
subject_id: PNN
004.run1
005.run1
006.run1
subject_id: SOA
007.run1
008.run1
009.run1
subject_id: TRU
001.run0
002.run0
003.run0
subject_id: VTI
012.run1
002.run1
003.run1
Bacteroides
Staphylococcus
Enterococcus
u(f__Enterobacteriaceae)
Parabacteroides
Corynebacterium
u(f__Erysipelotrichaceae)
u(o__Clostridiales)
Lactobacillus
[Ruminococcus]
Blautia
Prevotella
Bifidobacterium
Collinsella
[Eubacterium]
Bilophila
Streptococcus
u(f__Ruminococcaceae)
Dorea
Pseudoramibacter_Eubacte...
u(f__Rikenellaceae)
Coprococcus
Klebsiella
u(f__Enterococcaceae)
Eggerthella
u(f__Christensenellaceae)
u(f__Lachnospiraceae)
Serratia
u(f__Clostridiaceae)
u(f__Leuconostocaceae)
Lactococcus
Bulleidia
u(f__Coriobacteriaceae)
Catenibacterium
Christensenella
Granulicatella
Succinivibrio
u(f__[Barnesiellaceae])
Ruminococcus
Faecalibacterium
Phascolarctobacterium
SMB53
u(f__Peptostreptococcaceae)
Akkermansia
u(f__Planococcaceae)
u(o__RF39)
Oscillospira
Parvimonas
Clostridium
Haemophilus
