# Supplementary material for: Serum and fecal profiles of aromatic microbial metabolites reflect gut microbiota disruption in critically ill patients: a prospective observational pilot study
Source: Crit Care. 2020 Jun 8;24:312. doi: 10.1186/s13054-020-03031-0 (PMC7278238; doi:10.1186/s13054-020-03031-0)
Supplement: Supplementary file 2 — Additional file 2: Supplementary Figure 2. - Temporal dynamics of gut microbiome composition in the CCI patients. The heatmap of relative abundance at genus level is split into sections by subject, with rows of each section corresponding to consecutive time points. A snapshot from an online interactive report in Knomics-Biota. [file 13054_2020_3031_MOESM2_ESM.pdf]

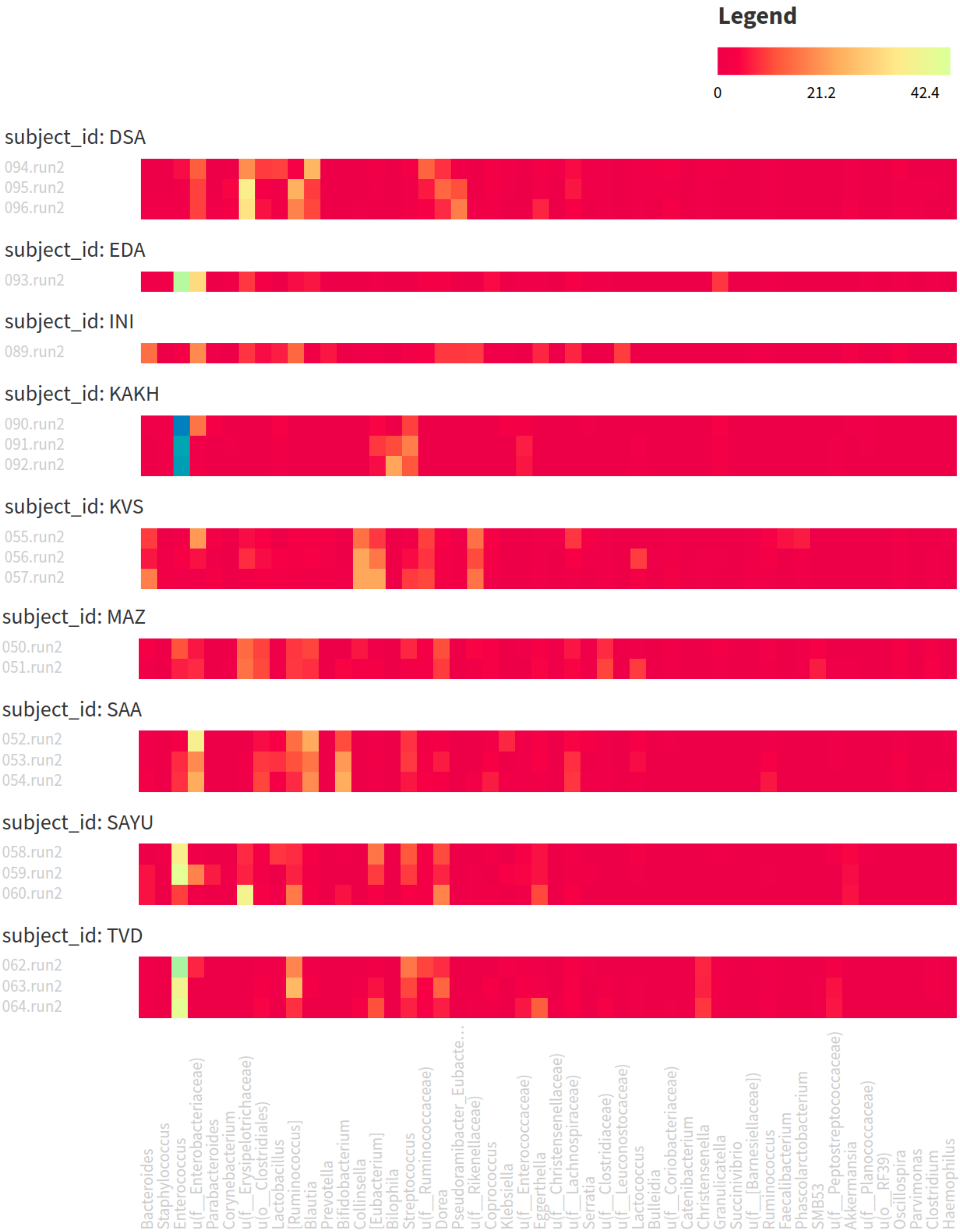
Legend
0
21.2
42.4
subject_id: DSA
094.run2
095.run2
096.run2
subject_id: EDA
093.run2
subject_id: INI
089.run2
subject_id: KAKH
090.run2
091.run2
092.run2
subject_id: KVS
055.run2
056.run2
057.run2
subject_id: MAZ
050.run2
051.run2
subject_id: SAA
052.run2
053.run2
054.run2
subject_id: SAYU
058.run2
059.run2
060.run2
subject_id: TVD
062.run2
063.run2
064.run2
Bacteroides
Staphylococcus
Enterococcus
u(f__Enterobacteriaceae)
Parabacteroides
Corynebacterium
u(f__Erysipelotrichaceae)
u(o__Clostridiales)
Lactobacillus
[Ruminococcus]
Blautia
Prevotella
Bifidobacterium
Collinsella
[Eubacterium]
Bilophila
Streptococcus
u(f__Ruminococcaceae)
Dorea
Pseudoramibacter_Eubacte...
u(f__Rikenellaceae)
Coprococcus
Klebsiella
u(f__Enterococcaceae)
Eggerthella
u(f__Christensenellaceae)
u(f__Lachnospiraceae)
Serratia
u(f__Clostridiaceae)
u(f__Leuconostocaceae)
Lactococcus
Bulleidia
u(f__Coriobacteriaceae)
Catenibacterium
Christensenella
Granulicatella
Succinivibrio
u(f__[Barnesiellaceae])
Ruminococcus
Faecalibacterium
Phascolarctobacterium
SMB53
u(f__Peptostreptococcaceae)
Akkermansia
u(f__Planococcaceae)
u(o__RF39)
Oscillospira
Parvimonas
Clostridium
Haemophilus
